# Estimated exercise‐induced maximal myocardial blood flow in untrained and endurance‐trained men

**DOI:** 10.1113/EP093307

**Published:** 2026-03-11

**Authors:** Ilkka Heinonen, Kari K. Kalliokoski, Juhani Knuuti, Marko S. Laaksonen

**Affiliations:** ^1^ Turku PET Centre University of Turku, Turku University Hospital, and Åbo Akademi University Turku Finland; ^2^ Department of Biomedical Engineering Huazhong University of Science and Technology Wuhan Hubei China; ^3^ Swedish Winter Sports Research Centre, Department of Health Sciences Mid Sweden University Östersund Sweden; ^4^ The UKK Institute for Health Promotion Research Tampere Finland

**Keywords:** adenosine, exercise, heart, maximal, men, myocardial blood flow, training

## Abstract

The heart critically depends on a continuous supply of oxygen, provided by myocardial blood flow (MBF), but reliable quantification of MBF during maximal exercise is not feasible in humans. We measured MBF at rest and during similar absolute (150 W) and relative (70% of ${\dot{V}}_{O_{2}\max}$) cycling exercise workloads in seven healthy untrained (UT) and seven healthy endurance‐trained (ET) men by positron emission tomography and [^15^O]H_2_O‐tracer. The maximum MBF was extrapolated using the linear relationship between cardiac work load and MBF. MBF was also measured during intravenous infusion of standard high dose adenosine known to cause maximal vasodilation in the myocardial vasculature. Maximal exercise‐induced MBF was calculated to be 5.0 ± 1.5 mL/g/min in UT and 4.5 ± 1.7 mL/g/min in ET (*p* = 0.6), which was 6.9‐fold and 6.5‐fold higher from their respective resting values. Adenosine‐induced MBF was 3.9 ± 1.4 mL/g/min in UT and 2.3 ± 0.9 mL/g/min in ET, being significantly lower than maximal exercise‐induced MBF in both groups. In addition, MBF was lower in the ET than in the UT group in both conditions. Myocardial vascular resistance responses and conclusions depended on whether the estimated maximal exercise calculations were based on mean arterial or systolic blood pressure estimations. This study thus demonstrates that estimated maximal MBF during exercise exceeds that achieved with adenosine infusion, and its increase from rest in humans may be higher than previously considered. Endurance training appears, however, not to increase peak MBF per gram of myocardium.

## Abstract (highlights)

- **What is the central question of this study?**
  What is the maximal myocardial blood flow (MBF) in humans?
- **What is the main finding and its importance?**
  Estimated maximal exercise‐induced MBF was significantly higher, over 6‐fold from the resting baseline, than the MBF achieved during high‐dose adenosine infusion. Further, endurance training does not increase peak MBF per gram of myocardium. These findings provide an increased understanding of myocardial oxygen supply during intense physical exertion.

## 1 INTRODUCTION

Proper function of the heart critically depends on a continuous supply of oxygen. As oxygen extraction in the human myocardium is already very high (60–80%) at rest, oxygen supply to the myocardium is provided almost solely by increased myocardial blood flow (MBF) (Duncker & Bache, 2008). Therefore MBF increases linearly with increasing heart rate or exercise intensity to meet the oxygen demands of the increasingly beating heart (Duncker & Bache, 2008). Blood flow may be even more important in well‐conditioned heart as myocardial oxygen extraction is higher both at rest and during exercise in endurance‐trained athletes than in untrained subjects (Heinonen et al., 2014). Thus, endurance athletes have limited capacity to increase myocardial oxygen extraction and they rely almost solely on increased MBF to deliver oxygen, especially during maximal exercise. In this respect it has also been proposed that MBF might be a limiting factor for maximal aerobic performance by affecting maximal cardiac output (Heinonen, 2025). However, there is no solid experimental evidence to support this view, while cardiac output and its regulation is clearly of importance in health and disease (Ainab et al., 2025; Drouin et al., 2025; Fischer et al., 2025b; Furst & Gonzalez‐Alonso, 2025; Lampkemeyer et al., 2025; Ogoh, 2025; O'Leary & Mannozzi, 2025; Sagmeister et al., 2025; Stohr, 2025).

Myocardial vascular function and capacity are typically assessed by measuring MBF during standard high‐dose intravenous infusion of adenosine, which is generally considered to cause maximal or near maximal myocardial vasodilation (Heusch, 2010). Cross‐sectional studies comparing trained and untrained subjects or training intervention studies mainly show that this adenosine‐stimulated MBF is not increased in the trained heart (Eskelinen et al., 2016; Hannukainen et al., 2007; Heinonen et al., 2008; Hildick‐Smith et al., 2000; Kalliokoski et al., 2002; Laaksonen et al., 2014; Radvan et al., 1997), although there is at least one exception (Toraa et al., 1999). There are, however, significant differences in many physiological parameters caused by adenosine‐ and exercise‐induced stress. For example, blood pressure and therefor perfusion pressure react differently in these stresses.

MBF during truly maximal exercise is challenging to measure due to technical difficulties. Consequently, in the present study we estimated exercise‐induced maximal MBF based on the data collected at rest and during two exercise intensities and compared the responses with the adenosine‐induced MBF by applying physiological modelling (Joyner, 2025). Furthermore, we investigated whether these responses differ between the untrained and endurance‐trained subjects.

## 2 METHODS

### 2.1 Ethical approval

The study followed the ethical standards of the *Declaration of Helsinki*, except for registration in a database. It was approved by the Ethical Committee of the Hospital District of Southwest Finland (ETMK 2/2002 1§29). Before taking part, all participants received both verbal and written information about the study design and protocol and provided written informed consent.

### 2.2 Participants

Seven untrained healthy males (age 25 ± 3 years, body mass index (BMI) 23 ± 2 kg/m^2^) and seven endurance‐trained male athletes (age 25 ± 3 years, BMI 21 ± 2 kg/m^2^) volunteered for the study. Untrained participants (UT, *n* = 7) were not involved in any systematic training during the study period or the year before it whereas the endurance athletes (ET, *n* = 7) had a training background from different endurance sports. The participants were instructed to maintain a regular diet but to avoid caffeinated drinks and alcohol during the last 24 h before the measurements. Subjects were also instructed to avoid any kind of strenuous physical activity 48 h before the experiments.

### 2.3 Study design

Peak oxygen consumption (${\dot{V}}_{O_{2}\mathrm{peak}}$), maximal heart rate (HR_max_) and power output (*W*_max_) were determined using a bicycle ergometer test 2 weeks prior to echocardiography and positron emission tomography (PET) studies. Echocardiographic measurements and PET studies were performed at least 3 h after a light breakfast. First, the left ventricular (LV) structure and function were assessed using two‐dimensional guided M‐mode echocardiography. Thereafter, a catheter was inserted in an antecubital vein for injection of radioactive tracer ([^15^O]H_2_O) and blood sampling, and the participants were then laid down on a vacuum base and were thereafter adjusted and fastened to the cycle ergometer in a semisupine position with three broad straps over the upper body, and inserted into the PET scanner, as reported previously (Laaksonen et al., 2007). Simultaneously, the subjects were allowed to familiarize themselves with the cycle ergometer. After a transmission scan, MBF was measured at rest and during adenosine‐infusion and two bicycle exercise loads (absolute same 150 W, and relative same 70% of *W*_max_) with [^15^O]H_2_O‐tracer.

### 2.4 Maximal bicycle ergometer test

${\dot{V}}_{O_{2}\mathrm{peak}}$, HR_max_ and *W*_max_ were determined by an incremental bicycle ergometer (Model 800S, Ergoline, Mijnhardt, The Netherlands) test in the upright sitting position with direct respiratory measurements 2 weeks before the PET procedure. ${\dot{V}}_{O_{2}}$ was measured using the Medikro 202 gas analyser (Medikro Oy, Kuopio, Finland) or MedGraphics cardiorespiratory diagnostic system (Medical Graphics Corp., St Paul, MN, USA) with 10‐s sampling frequency, and the criteria used to establish the ${\dot{V}}_{O_{2}\mathrm{peak}}$ were a plateau in ${\dot{V}}_{O_{2}}$ despite increase in working intensity and a respiratory quotient of > 1.1. The mean of the three highest consecutive ${\dot{V}}_{O_{2}}$ values within a 30‐s time frame was considered as ${\dot{V}}_{O_{2}\mathrm{peak}}$.

### 2.5 Echocardiography

The participants rested first for at least 15 min in a left lateral decubitus position and were thereafter examined for LV end‐diastolic and end‐systolic wall thicknesses, volumes and dimensions using two‐dimensional M‐mode echocardiography (Acuson 128XP, Acuson, Mountain View, CA, USA). LV mass was calculated using the Penn convention.

### 2.6 Measurement and calculation of MBF

A transmission scan for the correction of photon attenuation was performed when participants were resting in the ECAT 931/08 tomograph scanner (Siemens/CTI, Knoxville, TN, USA). Thereafter, [^15^O]H_2_O (half‐life 2.05 min) was injected intravenously for 2 min, and dynamic PET scanning was performed for 6 min (resting baseline); 15 min from the start of the baseline MBF measurement, a similar PET scan was repeated during 6 min of intravenous administration of adenosine (140 µg kg^−1^ min^−1^) known to induce maximal myocardial vasodilation. Thereafter, participants started a bicycle exercise for 25 min during which MBF was measured at the intensities of 150 W and 70% of *W*_max_. All PET data were corrected for dead time, decay and measured photon attenuation, and images were processed with the iterative reconstruction algorithm.

Regions of interest (ROIs) were drawn on four representative transaxial mid‐LV slices covering the anterior and lateral myocardial walls (whole) of the LV. ROIs were drawn on the images obtained at rest and were copied to the images obtained during adenosine infusion and both exercise intensities. The input function was obtained from the LV time–activity curve. Myocardial perfusion was then calculated with the previously introduced method employing the single‐compartment model (Heinonen et al., 2008; Kalliokoski et al., 2002; Koivula et al., 2024; Laaksonen et al., 2007, 2014), and mean MBF values of four transaxial slices at rest, during adenosine infusion and during both exercise intensities were obtained.

### 2.7 Exercise during PET and prediction of maximal MBF

After baseline and measurements during adenosine infusion, participants started the exercise, using a cycle ergometer (Bosch ERG 555, Geschäftsbereich Elektronik, Germany). First, subjects cycled for 2 min at a level of 40% of measured *W*_max_ (ET 133 ± 5 vs. UT 93 ± 6 W; *p* < 0.001). Thereafter, the exercise intensity was increased to 150 W, and after 3 min of exercise MBF was measured. This absolute exercise intensity corresponded to the relative intensity of 42 ± 2% of *W*_max_ for ET and 59 ± 4% of *W*_max_ for UT subjects. Immediately after the MBF measurement at the 150‐W exercise level, the exercise intensity was decreased back to the level of 40% of *W*_max_ for 5 min. Thereafter, the exercise intensity was increased to 70% of *W*_max_ (ET 250 ± 14 vs. UT 180 ± 13 W; *p* < 0.001) for 9 min to repeat the MBF measurement. According to a previous report, to equalize the relative submaximal exercise load during the PET experiment in a semisupine position, the workload derived during the upright bicycle exercise was individually multiplied by 0.93 (Laaksonen et al., 2007). The pedalling rate was 50–55 rpm to minimize body motion during PET scanning.

Prior to calculation of maximal predicted MBF, maximal rate–pressure product (RPP) was first extrapolated from the linear relationship between systolic blood pressure (SBP) and HR, using the measured HR_max_ as an end point individually for each participant (average Pearson correlation coefficient 0.96 ± 0.06). Thereafter, the maximal MBF was predicted using this maximal RPP and the linear relationship between RPP and MBF at rest, absolute and relative exercise levels (Figure A2, average Pearson correlation coefficient 0.93 ± 0.09).

### 2.8 Other measurements and calculations

HR during the PET experiment was automatically recorded with an ECG recorder (MAC 5000; GE Marquette Medical Systems, Milwaukee, WI, USA). Blood pressure (SBP and diastolic, DBP) was monitored with an automatic oscillometric BP analyser (Omrom, Tokyo, Japan) throughout the entire PET procedure. Myocardial vascular resistance (MVR) was calculated by dividing mean arterial pressure (MAP) by the respective MBF value. MAP was calculated as [(2 × diastolic) + systolic]/3. As systolic blood pressure is markedly increased and diastolic time is markedly reduced during maximal exercise and MAP stems largely from diastolic blood pressure, which is also difficult to predict at maximal exercise, MVR during estimated maximal exercise was also calculated by dividing systolic arterial pressure by the respective MBF value.

### 2.9 Statistical analysis

In cases where there was only one time point, group differences were analysed by Student's *t*‐test. Multilevel models for repeated measures were used to analyse the effects of the different conditions. In all the models, main factors were time and group, and group‐by‐time interaction (group × time) was used to evaluate whether mean changes over conditions differed between the groups. Tukey–Kramer method was used to adjust for multiple comparisons. The data are presented in terms of mean standard ± deviation (SD). A significance level of 5% (two‐tailed) was employed for all statistical analyses. Analyses were performed with SAS 9.4 (SAS Institute Inc., Cary, NC, USA). *n* = 7 in both groups in all data.

## 3 RESULTS

**FIGURE A1:**
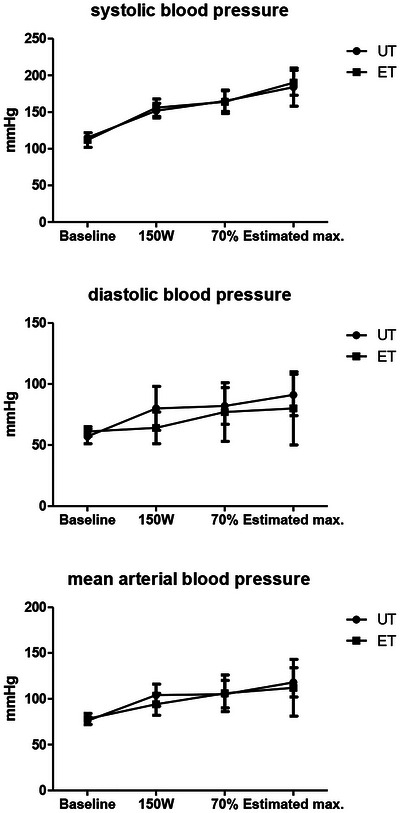
Blood pressure responses to exercise. ET, endurance‐trained; Max, maximal; UT, untrained.

**FIGURE A2:**
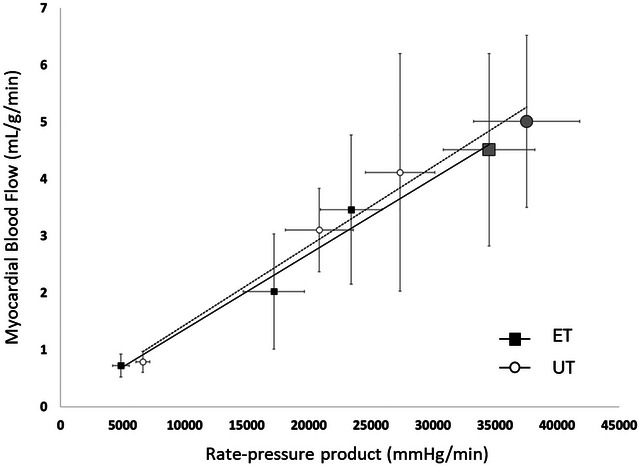
Relationship between rate–pressure product and myocardial blood flow (MBF), which formed the basis to calculate the estimated maximal exercising MBF values. ET, endurance‐trained; UT, untrained. The linear lines represent the regression between these two variables (continuous line ET, *y* = 0.0001*x* + 0.0352, *R* ^2^ = 0.98; dashed line UT, *y* = 0.001*x* + 0.0442, *R* ^2^ = 0.98). The grey colour represents the estimated maximal values.

Some of the data collected for this study have been published earlier (Laaksonen et al., 2007) and therefore, only the most relevant parts of those results will be presented here. HR, DBP and SBP as well as RPP values at rest and during exercise and adenosine infusion are shown in Table 1. We have also previously reported that in line with reduced resting HR, this endurance athlete population documented clear athlete's heart as LV mass (193 ± 20 g in UT and 309 ± 56 g in ET, *p* < 0.001) and stroke volume were significantly higher in the ET group (93 ± 8 mL in UT and 113 ± 13 mL in ET, *p* = 0.004), while systolic and diastolic functions were normal (Laaksonen et al., 2007). Further, while resting MBF did not differ between the groups, the ET group had lower MBF during the similar absolute exercise load, but similar MBF during the same relative exercise intensity workload (Laaksonen et al., 2007). Maximal heart rate during the incremental upright bicycle exercise test until exhaustion was 195 ± 13 bpm in the UT and 182 ± 10 bpm in the ET group (*p* = 0.057 between the groups). The responses of blood pressures and estimated maximal blood pressures and the relationship between estimated RPP and MBF are shown in the Appendix (Figures A1 and A2). *n* = 7 in both groups in all data.

**TABLE 1:** Hemodynamic measures at rest and during sub‐maximal exercise, and adenosine infusion.

		**Resting baseline**	**150 W Exercise**	**70 % Exercise**	**ANOVA**	**Adenosine infusion**
HR, bpm	UT	58 ± 6	136 ± 12	162 ± 10	group p < 0.001	84 ± 17
	ET	44 ± 6	110 ± 12	143 ± 9	time p < 0.001	56 ± 10
					group*time p = 0.230	
DBP,	mmHg	57 ± 6	80 ± 18	82 ± 15	group p = 0.475	51 ± 3
UT		61 ± 4	64 ± 13	77 ± 24	time p < 0.001	58 ± 4
	ET				group*time p = 0.114	
SBP,	mmHg	115 ± 3	152 ± 10	153 ± 24	group p = 0.492	110 ± 3
UT		112 ± 10	156 ± 12	164 ± 24	time p < 0.001	116 ± 12
	ET				group*time p = 0.873	
RPP,	mmHg/min	4421 ± 487	14261 ± 2237	17184 ± 3125	group p < 0.01	6008 ± 1507
UT		3375 ± 361	10459 ± 1959	15120 ± 2922	time p < 0.001	4295 ± 738
	ET				group*time p = 0.723	

*Note*: Data are means ± SD (*n* = 7 for both UT and ET). Resting and exercising heart rates and blood pressure and RPP values at rest and during submaximal exercise and their statistics previously published (Laaksonen et al., 2007). Abbreviations: DBP, diastolic blood pressure; ET, endurance trained; HR, heart rate; RPP, rate–pressure product (HR × systolic blood pressure); SBP, systolic blood pressure; UT, untrained.

**FIGURE 1:**
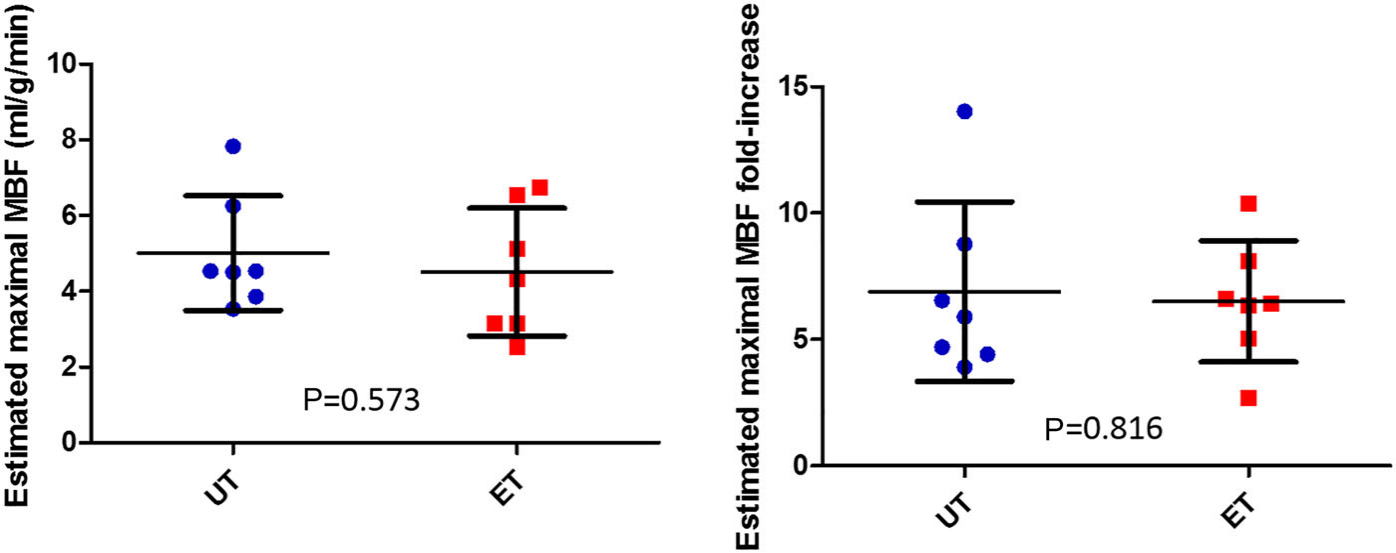
Myocardial blood flow (MBF) during estimated maximal exercise and its fold‐increase from resting baseline in untrained (UT, *n* = 7) and endurance‐trained (ET, *n* = 7) men. Error bars shown are ± SD.

**FIGURE 2:**
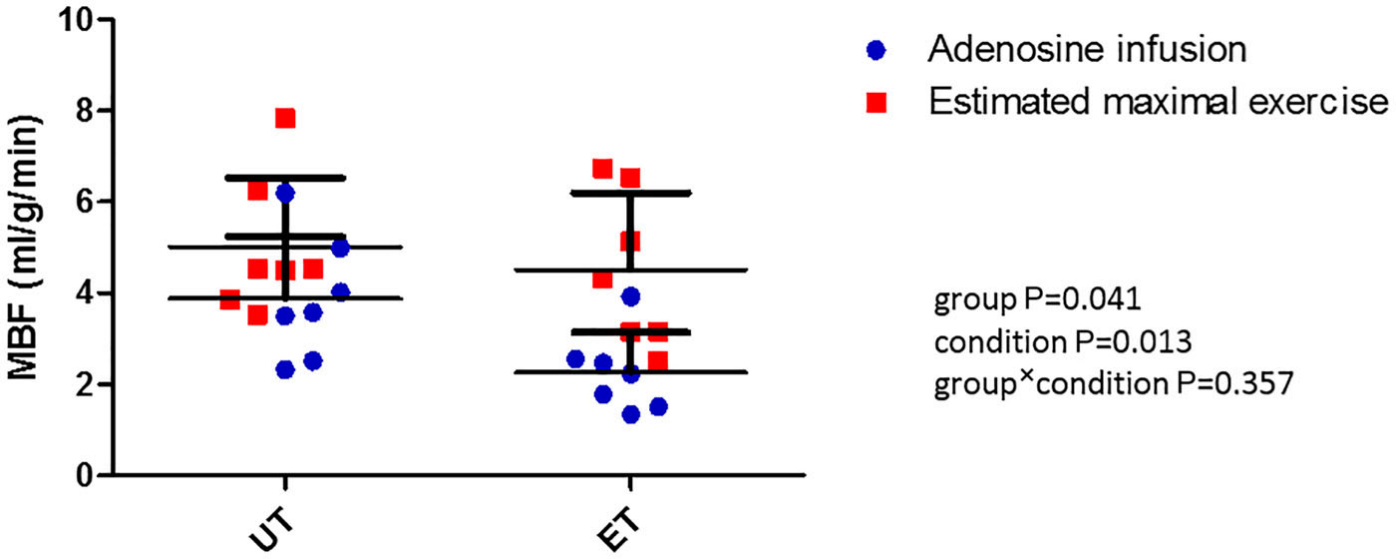
Myocardial blood flow (MBF) during high‐dose adenosine infusion and during estimated maximal exercise in untrained (UT, *n* = 7) and endurance‐trained (ET; *n* = 7) men. Error bars shown are ± SD.

Maximal, estimated exercise‐induced MBF was 5.0 ± 1.5 mL/g/min in UT and 4.5 ± 1.7 mL/g/min in ET (*p* = 0.573 between the groups), which was 6.9 ± 3.5‐fold and 6.5 ± 2.4‐fold higher (*p* = 0.816) than their respective resting values (Figure 1). Adenosine‐induced MBF was 3.9 ± 1.4 mL/g/min in UT and 2.3 ± 0.9 mL/g/min in ET, being significantly lower than predicted maximal MBF during exercise in both groups (Figure 2). In addition, the group difference was significant, the MBF being lower in ET (Figure 2).

**FIGURE 3:**
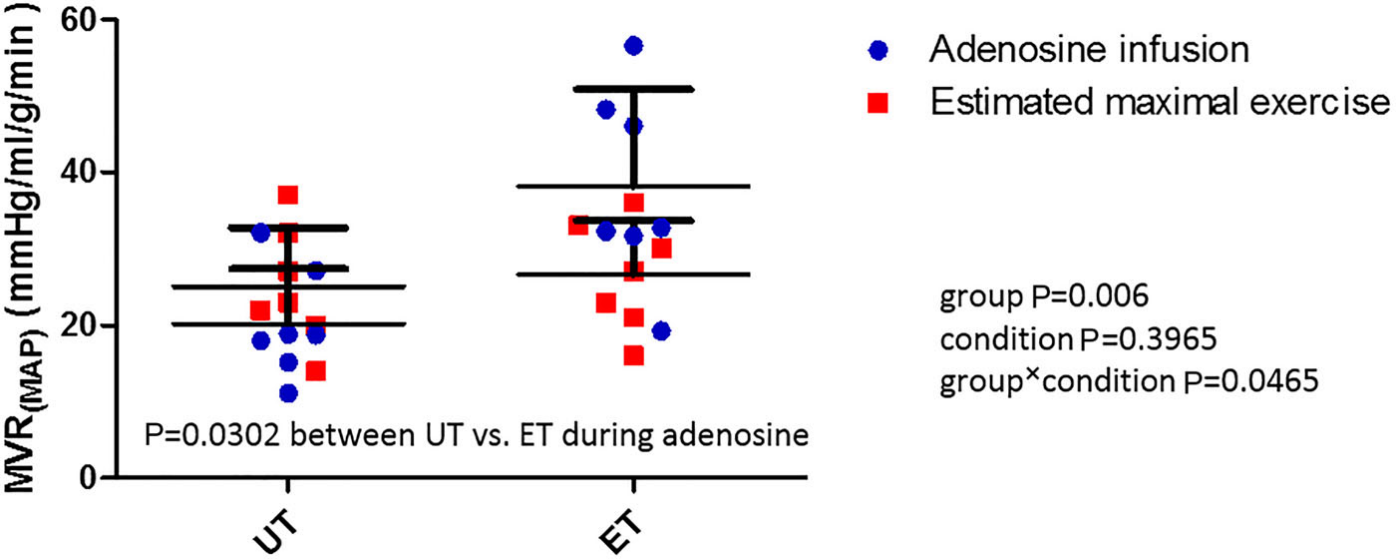
Myocardial vascular resistance (MVR) based on mean arterial blood pressure during high‐dose adenosine infusion and during estimated maximal exercise in untrained (UT, *n* = 7) and endurance‐trained (ET; *n* = 7) men. Error bars shown are ± SD.

**FIGURE A3:**
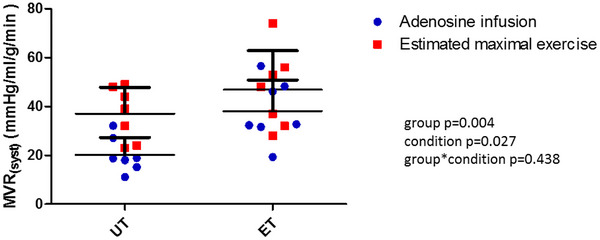
Myocardial vascular resistance (MVR) during high‐dose adenosine infusion and during estimated maximal exercise in untrained (UT) and endurance‐trained (ET) men. Error bars shown are ± SD.

MAP was 76 ± 4 mmHg in UT and 78 ± 6 mmHg in ET and tended to be reduced by adenosine infusion (71 ± 6 mmHg in UT and 77 ± 5 mmHg in ET, *p* = 0.050). MAP during estimated maximal exercise was 118 ± 15 mmHg in UT and 112 ± 31 mmHg in ET and there was a significant condition effect for the adenosine infusion (*p* < 0.0001, group *p* = 0.931, group × conidition *p* = 0.349). When MVR during both adenosine and estimated maximal exercise was calculated based on the MAP, there was a significant group × condition interaction in the responses, so that MVR was significantly higher in ET during adenosine infusion (Figure 3). However, when MVR during estimated maximal exercise was calculated by estimated systolic blood pressure, it decreased significantly from rest to both adenosine‐ and estimated exercise‐induced maximal vasodilation, and the MVR during the adenosine infusion was significantly lower than that of the estimated maximal exercise between these conditions (Figure A3). However, MVR was significantly higher in ET compared to UT in both conditions (Figure A3).

Estimated maximal exercise‐induced total left ventricular (LV) MBF, and thus MBF per gram of myocardium multiplied by LV mass, did not differ between the groups (total LV MBF 972 ± 327 mL/min in UT and 1398 ± 585 mL/min in ET, *p* = 0.12). Finally, explorative correlations between fitness measures and MBF and MVR are shown in the Appendix.

## 4 DISCUSSION

This study sought to provide novel insights into human myocardial perfusion dynamics by estimating maximal exercise‐induced myocardial blood flow (MBF) and comparing it with adenosine‐induced MBF in both untrained (UT, *n* = 7) and endurance‐trained (ET, *n* = 7) men. Importantly, the study design allowed for a physiologically relevant extrapolation of peak MBF based on a robust linear relationship between RPP and measured MBF during submaximal cycling, while also offering the first direct comparison between pharmacologically and exercise‐induced coronary vasodilation in athletes versus controls.

**FIGURE A4:**
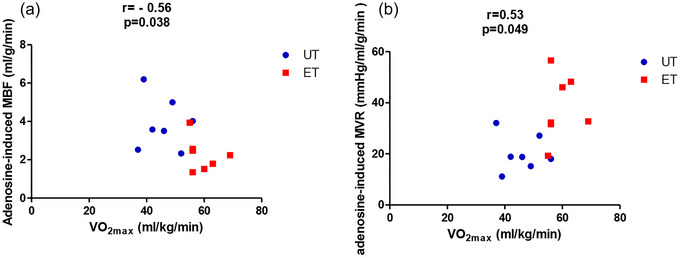
Correlations between maximal oxygen uptake (${\dot{V}}_{O_{2}\max}$) and adenosine‐induced myocardial blood flow (MBF, a) and myocardial vascular resistance (MVR, b) in the whole study population. ET, endurance‐trained; UT, untrained.

A key finding was that the estimated maximal exercise‐induced MBF was significantly higher, over 6‐fold from resting baseline, than the MBF achieved during high‐dose adenosine infusion in both groups. In addition, both adenosine and exercise‐induced maximal MBF were significantly lower in endurance‐trained myocardium. This result challenges the traditional assumption that the exercise‐induced increase in MBF is lower than the MBF during pharmacological stress such as adenosine infusion, which has been generally considered to represent a proxy for maximal vasodilation. Notably, while adenosine is a potent coronary vasodilator, it does not replicate the haemodynamic conditions of maximal exercise, particularly the elevated perfusion pressure (Laughlin, 1987). Further, in addition to exercise, the adenosine response is also significantly part endothelium dependent (Buus et al., 2001). Of note, while adenosine‐induced blood flow in the skeletal muscles correlates positively with the whole body ${\dot{V}}_{O_{2}\max}$ (Heinonen et al., 2010), adenosine‐induced MBF correlated negatively (Figure A4) with the ${\dot{V}}_{O_{2}\max}$ in the present study, while estimated exercise‐induced maximal MBF did not.

Another novel observation was that endurance training did not enhance the absolute peak MBF, despite significantly different cardiac structures and resting haemodynamics. This suggests that training‐induced cardiac adaptations may improve mechanical efficiency (e.g., stroke volume and LV mass), but do not necessarily enhance the maximal perfusion capacity of the myocardium per gram of tissue. When normalized to LV mass, total MBF tended to be higher in ET, but this difference did not reach statistical significance. The implication is that structural and functional adaptations of the athlete's heart may support elevated cardiac output without requiring proportionally higher myocardial perfusion per gram of tissue.

In regards to minimal myocardial vascular resistance (MVR), our results significantly depended on whether they were calculated based on the predicted MAP or the systolic blood pressure during estimated maximal exercise. When it was calculated with the MAP, similarly as during adenosine infusion to allow direct comparison, there was no condition effect and MVR was higher in the ET than UT group during adenosine infusion, but not during estimated maximal exercise (Figure 3). We do not have a clear explanation why vascular resistance remained higher in the ET than UT group during the adenosine infusion condition, which should cause minimal vascular resistance also in endurance‐trained heart. One interpretation is that the ET heart retains a greater vasodilatory reserve, possibly due to chronic adaptations in vascular tone regulation or a shift in perfusion–perfusion pressure dynamics. This preserved reserve might serve as a protective mechanism, allowing athletes to accommodate further increases in demand or to mitigate ischaemic risk under extreme physiological stress. However, the functional significance of this finding remains speculative and warrants further investigation and is further complicated by the fact that estimated maximal exercise MVR was similar between the groups, suggesting depleted vasodilatory reserve during exercise. Further, when MVR during maximal exercise was calculated with systolic blood pressure (Figure A3) as during exercise the diastolic time is markedly reduced and the contribution of systole increases, MVR was higher in endurance‐trained heart during both of these two vasodilation conditions, suggesting that endurance‐trained heart had not yet depleted its vasodilation reserve even during maximal exercise. Finally, the MVR during estimated maximal exercise based on systolic blood pressure was significantly lower during adenosine infusion than during the estimated maximal exercise in both groups, suggesting that adenosine could have caused larger vasodilation in myocardial vasculature than estimated maximal exercise. However, in the absence of direct measurements of true pressures, particularly pressure–flow measurements, no firm conclusions can be drawn to judge what really happens with MVR, or whether MAP or systolic blood pressure should be used to calculate MVR during maximal exercise in these human investigations without invasive measurements. Therefore, the focus in the present study should be on the MBF results.

True maximal MBF during exercise has remained to be measured in humans, although it is sometimes claimed that myocardial or coronary blood flow was measured during maximal exercise (Grubbstrom et al., 1991). However, in these studies, HR has been below 170 bpm in young healthy males, which indicates that exercise has likely not been maximal and is comparable to the exercise level of 70% of *W*_max_  in the present study according to the measured HR levels. Moreover, the measured HR_max_ was above 180 bpm in ET and 195 bpm in UT in the present study.

MBF during truly maximal exercise is challenging to measure due to technical difficulties and body movement during maximal effort, especially if imaging techniques, including ultrasound (Fischer et al., 2025a), are used. Therefore, we did not aim to measure MBF during truly maximal effort, but relied on its prediction based on the linear relationship between RPP measured during submaximal exercise intensities when MBF was also measured, and when there was also no substantial movement of the upper body during semi‐supine bicycle exercise. A methodological strength of the present study lies in the use of PET imaging with [^15^O]H_2_O and a validated model for MBF quantification under controlled submaximal exercise conditions. While direct measurement of MBF during maximal exertion is technically infeasible due to motion artifacts and safety concerns, the extrapolative approach used here is grounded in well‐established physiological relationships, yielding a robust estimate of peak perfusion capacity. Nonetheless, it should be acknowledged that this extrapolation may underestimate true maximal MBF, particularly in the ET individuals capable of achieving higher blood pressures (Caselli et al., 2016) than those observed in the semi‐supine cycling protocol. Further, as this study was performed only in white males and as the physiological responses may differ particularly between sexes (Ansdell et al., 2020; Koivula et al., 2025), but also depending on age and race, further studies on the topic are required in more dispersed study populations.

### 4.1 Conclusion

In conclusion, this study demonstrates that estimated maximal MBF during exercise exceeds that achieved with adenosine infusion. Therefore, MBF and its increase from rest in humans may be higher than previously considered. Endurance training does not, however, appear to increase peak MBF per gram of myocardium. These findings provide an increased understanding of myocardial oxygen supply during intense physical exertion.

## AUTHOR CONTRIBUTIONS

All authors have participated in study planning, performing, and writing the manuscript. All authors have read and approved the final version of this manuscript and agree to be accountable for all aspects of the work in ensuring that questions related to the accuracy or integrity of any part of the work are appropriately investigated and resolved. All persons designated as authors qualify for authorship, and all those who qualify for authorship are listed.

## CONFLICT OF INTEREST

None declared.

## Data Availability

Data will be available on a reasonable request.

## Appendix

Blood pressure responses to exercise are shown in the Figure A1 and the relationship between ratepressure product and myocardial blood flow is shown in the Figure A2. As the diastolic time markedly shortens and the diastolic blood pressure is hard to measure and estimate during maximal exercise, and the contribution to systolic blood pressure increases towards maximal exercise, although myocardial blood flow still occurs during diastole, we calculated minimal myocardial vascular resistance (MVR) during estimated maximal exercise also based on systolic blood pressure, which is shown in the Figure A3. MVR during the adenosine infusion was significantly lower than that of the estimated maximal exercise between these conditions (Figure A3). However, MVR was significantly higher in the ET compared to the UT in both conditions (Figure A3). Adenosine‐induced MVR is based on the MAP.

### Correlations between the measured key variables

Adenosine‐induced MBF or MVR did not correlate with the estimated exercise‐induced maximal MBF or MVR, respectively (data not shown, *p* > 0.53 in all correlations), nor did estimated exercise‐induced maximal MBF or MVR correlate with the ${\dot{V}}_{O_{2}\max}$ or *W*_max_ (*r* = 0.69, *p* = 0.09 for maximal MBF and ${\dot{V}}_{O_{2}\max}$ in the UT group, *p* > 0.28 in all other correlations, either amongst all the subjects or in each group separately). However, adenosine‐induced MBF correlated negatively and MVR positively with ${\dot{V}}_{O_{2}\max}$ (Figure A4). There was also a trend for adenosine‐induced MBF (*r* = −0.49, *p* = 0.07) and MVR (*r* = 0.53, *p* = 0.05) to correlate with *W*_max_ amongst the all subjects.
